# Editorial: Multimodality Imaging in Valvular Heart Disease

**DOI:** 10.3389/fcvm.2021.708889

**Published:** 2021-07-19

**Authors:** Thomas A. Treibel, Jonathan Bennett, João L. Cavalcante

**Affiliations:** ^1^Institute for Cardiovascular Sciences, University College London, London, United Kingdom; ^2^Barts Health NHS Trust and University College London, London, United Kingdom; ^3^Lewisham and Greenwich NHS Trust, London, United Kingdom; ^4^Minneapolis Heart Institute at Abbott Northwestern Hospital, Minneapolis, MN, United States

**Keywords:** cardiac computed tomographic imaging, cardiac magnetic resonance imaging, echocardiography, valvular heart disease, multi-modality imaging

Valvular heart disease (VHD) is a major cause of morbidity and mortality worldwide which with the population aging in the Western world has resulted in a 45% concurrent increase in non-rheumatic VHD (1). VHD poses not only a significant challenge from a structural and physiological perspective, but also from a diagnostic perspective with timing of referral, intervention ever more challenging in a multi-morbid population.

Our understanding of VHD is rapidly evolving expand the framework more broadly by considering the consequences of VHD into cardiac structure, function and remodeling. All of this has been sped along by the adoption of a multi-modality imaging (MMI) approach. While echocardiography remains the cornerstone for the initial diagnosis and evaluation, the modern heart valve specialist must also be versed in additional imaging modalities, which can aid in the diagnosis, patient selection and treatment planning. This comprehensive evaluation in VHD provided by cardiac imaging is essential in diagnosis and mechanism of valve dysfunction, severity quantification, procedural selection, intra-procedural guidance, and follow up for outcome and complications. This Frontiers topic collection collates some exemplars of recent advances in multimodality imaging and provides the clinician with the knowledge to deliver an individualized approach to their patients with VHD.

Discordance between aortic stenosis (AS) severity and gradients are not uncommon, occurring in up to 40% of patients. Guzzetti et al. update us on the nuances of assessing patients with low-gradient AS. In this situation transthoracic echocardiography (TTE) is the foundation of assessment; MMI has an increasing role to play to appreciate current paradigm that AS is a disease of valve and myocardium. They evaluate the established role of cardiac computed tomography (CCT) for valve calcification quantification, and the evolving role cardiac magnetic resonance imaging (CMR) for the myocardial assessment for detecting those who may benefit from current non-guideline-based intervention.

The management of AS has been radically changed since the advent of transcatheter aortic valve replacement (TAVR). Experience and success in this field has spurred on research into mitral valve intervention where there is a vast burden of morbidity but limited evidence base. The advent of MitraClip (2) and its mortality benefit has renewed interest in secondary mitral regurgitation (SMR) but morbidity and mortality remain high. Sharma et al. describe the role of echocardiography in assessment with the growing role of CMR for reliable, reproducible chamber assessment, providing optimal multi-modality assessment to improve the outcome of these patients.

Salvatore et al. examine the current evidence base for selecting and monitoring the optimal candidate for transcatheter edge-to-edge repair (TEER) with MitraClip device (Abbott, Menlo Park, CA). They highlight the evolving role of stress echocardiography being a valuable tool to optimize timing of intervention, and of cardiac magnetic resonance (CMR) in the comprehensive evaluation of SMR severity, and myocardial fibrosis (3). CMR evaluation should be considered particularly when transcatheter intervention trials in SMR (2, 4) have not been consistent in their outcome which in part might be due to population heterogeneity and underlying ventricular disease.

Beyond TEER, transcatheter mitral valve replacement (TMVR) is a rapidly progressing field, with TMVR increasingly used for failed mitral prosthesis (valve-in-valve), as well as valve-in-ring, and valve-in-MAC (mitral annular calcification). Additionally, paravalvular leak (PVL) closure—both of surgical and percutaneous prostheses—is an increasing indication for transcatheter intervention. TMVR and PVL are complex anatomically and physiologically, and therefore require multi-modality imaging at all stages to define anatomy and mechanism, procedural planning, and intraprocedural guidance. Garcia-Sayan et al. summarize the key role of CCT in predicting left ventricular outflow tract obstruction and percutaneous access planning, and provide a multi-modality imaging framework for intraprocedural guidance to maximize procedural outcomes.

Tayal et al. investigate the evolving application of multimodality to patients with mitral valve prolapse (MVP) and risk of ventricular arrhythmia. A disease entity described by Barlow (5) more than 50 years ago but for which an evidenced based approach to risk assessment of arrhythmia and sudden cardiac death continues to be lacking. The authors explore mechanical dyssynchrony and its implication in MVP, and discuss the novel use of echo tissue Doppler and contemporary strain imaging on the hypermobility of the myocardium. They highlight the use of CMR that reveals unique patterns of fibrosis in MVP with particular attention to the papillary muscles, which exhibit excessive movement and traction.

While CMR is now the gold standard for myocardial volumes, function and myocardial scar, its utility in the hemodynamic assessment of VHD is becoming increasingly evident. Fidock et al. examine the role of CMR in quantifying mitral regurgitation (MR), where CMR can offer not only precise ventricular assessment but also accurate quantification of the MR severity—particularly important in asymptomatic patients. Echocardiography, predominantly 3D transesophageal echocardiography, is the established benchmark but with the advent of 4D flow CMR, the multi-directional and dimensional regurgitation in MR can now be fully appreciated and quantified. 4D CMR involves phase contrast acquisition with velocity encoding in three-dimensions and time resolved. This systematic review compares 4D CMR to established echo techniques and older 2D CMR methods adding a reliable and reproducible technique in what can be a difficult surveillance and timing of intervention process.

4D flow by CMR is additionally providing insight into the mechanism of aortopathy in the bicuspid aortic valve (BAV) disease, the most common congenital lesion. BAV confers a chronic elevated risk of aortic dissection even after valve intervention (which 50% of patients require in their lifetime). Cave et al. describe how 4D flow can visualize and quantify the eccentric aortic flow in BAV patients and link this to the risk of developing aortopathy. They highlight recent insights into the flow dynamics of the various surgical techniques and devices available in this population (e.g., Ross autografts, mechanical and bioprosthetic valves).

We believe that this collection of multi-modality imaging applications in valvular heart disease offers the reader a contemporary range of exciting exemplars in this rapidly progressing field. Incorporation of multi-modality imaging provides valuable insights into mechanisms, which should be part for future randomized control trial to improve the outcomes of patients burdened with VHD. We hope that this collection will stimulate clinicians utilize an integrative approach to VHD in their practice.

## Author Contributions

This Editorial was conceived and written by all authors.

## Conflict of Interest

The authors declare that the research was conducted in the absence of any commercial or financial relationships that could be construed as a potential conflict of interest.
